# The economic burden of infertility treatment and distribution of expenditures overtime in France: a self-controlled pre-post study

**DOI:** 10.1186/s12913-022-07725-9

**Published:** 2022-04-15

**Authors:** B. Bourrion, H. Panjo, P.-L. Bithorel, E. de La Rochebrochard, M. François, N. Pelletier-Fleury

**Affiliations:** 1 INSERM, Université Paris-Saclay, UVSQ, CESP, Bâtiment 15/16 Inserm, Hôpital Paul Brousse, 16 Avenue Paul Vaillant Couturier, 94807 Villejuif Cedex, France; 2 Département de Médecine Générale, Faculté Des Sciences de La Santé Simone Veil, UVSQ, 2 Avenue de la Source de la Bièvre, 78180 Montigny le Bretonneux, France; 3 INED, 9 Cours des Humanités, 50004, 93322 Aubervilliers Cedex, CS France

**Keywords:** Costs and Cost Analysis, Infertility, Reproductive technique, assisted, Ovarian stimulation

## Abstract

**Background:**

Recent cost studies related to infertility treatment have focused on assisted reproductive technologies. None has examined lower-intensity infertility treatments or analyzed the distribution of infertility treatment expenditures over time.

The Purpose of the study was to analyse the size and distribution of infertility treatment expenditures over time, and estimate the economic burden of infertility treatment per 10,000 women aged 18 − 50 in France from a societal perspective.

**Methods:**

We used French National individual medico-administrative database to conduct a self-controlled before-after analytic cohort analysis with 556 incidental women treated for infertility in 2014 matched with 9,903 controls using the exact matching method. Infertility-associated expenditures per woman and per 10,000 women over the 3.5-year follow-up period derived as a difference-in-differences.

**Results:**

The average infertility related expenditure per woman is estimated at 6,996 (95% CI: 5,755–8,237) euros, the economic burden for 10,000 women at 70.0 million (IC95%: 57.6–82.4) euros. The infertility related expenditures increased from 235 (IC95%: 98–373) euros in semester 0, i.e. before treatment, to 1,509 (IC95%: 1,277–1,741) euros in semester 1, mainly due to ovulation stimulation treatment (47% of expenditure), to reach a plateau in semesters 2 (1,416 (IC95%: 1,161–1,670)) and 3 (1,319 (IC95%: 943–1,694)), where the share of expenses is mainly related to hospitalizations for assisted reproductive technologies (44% of expenditure), and then decrease until semester 6 (577 (IC95%: 316–839) euros).

**Conclusion:**

This study informs public policy about the economic burden of infertility estimated at 70.0 million (IC95%: 57.6–82.4) euros for 10,000 women aged between 18 and 50. It also highlights the importance of the share of drugs in infertility treatment expenditures. If nothing is done, the increasing use of infertility treatment will lead to increased expenditure. Prevention campaigns against the preventable causes of infertility should be promoted to limit the use of infertility treatments and related costs.

**Supplementary Information:**

The online version contains supplementary material available at 10.1186/s12913-022-07725-9.

## Background

Estimates of infertility prevalence and treatment seeking can vary according to the way infertility and medical care for infertility are defined and assessed [1], but in all cases the individual and societal burden of infertility worldwide is high [2]. This increase in the prevalence of infertility in women affects all countries regardless of their socio-demographic index (SDI). Compared to global prevalence, prevalence is higher in low- and middle-income countries where access to infertility treatment is limited by a cost barrier [2–4]. Although the prevalence is lower in countries with a high SDI, they are experiencing the greatest increase. In a prevalent representative cohort of women who were selected from the general population in France, it was estimated that 9% of women of childbearing age consult for fertility troubles after 12 months of unprotected sexual intercourse and 12% after 24 months [5]. Moreover, the use of infertility treatments is expected to increase due to ongoing sociodemographic and lifestyle changes. The age of birth of the first child, recognised as a major risk factor for infertility, continues to rise because of longer education, difficulties in accessing stable employment, and the fragility of marital and non-marital unions [6, 7]. Between 2010 and 2016, the share of births among women aged 35 years or older increased from 19.3% to 21.3% [7]. In parallel, environmental changes such as increased tobacco and alcohol consumption and rising obesity threaten fertility [8–10]. Very recently, Messaoud et al. estimated that 1.25% (IC95%: 1.23 − 1.27) of French women aged 20 to 49 years were treated for infertility each year [9]. In this longitudinal study, the mean age of treated women was 33.0 years in 2008 and 33.7 years in 2017. Over the decade, infertility treatment use increased by 23.9% (IC95%: 14.66% − 33.74%) among women aged 34 years or older.

In Europe in recent years, the use of assisted reproductive technologies (ART) has steadily increased [10]. ART refers to all the clinical and biological practices that enable In-Vitro-Fertilization (IVF), the conservation of eggs and sperm, germinal tissue and embryos, and the transfer of embryos and artificial insemination. In 2018, in France, nearly 150,000 ARTs have been performed leading to more than 25,000 births, representing more than 3% of the children [11, 12]. However, ART represents a small part, in terms of volume, of infertility management. A vast majority of women (87%) uses hormonal stimulation compared to 31% for IVF and 24% for artificial insemination [13].

Given this context of demographic changes, and to inform public policies, it seemed relevant to study the economic burden of infertility treatments and the distribution of expenditures. There are few recent articles in the literature that looked at the expenditures per woman treated for infertility. Their results vary from one study to another depending on the time horizon chosen, the perspective adopted, and the expenditures taken into account. In addition, the analysis of the distribution of infertility treatment expenditures over time is not addressed in these papers [14–16]. For example, Katz et al. focused on women treated for infertility, whether they received IVF or lower-intensity infertility treatments [14]. This was an 18-month prospective cohort study involving 312 women who consulted for infertility in 8 reproductive endocrinology practices in California. Following a societal perspective, the authors estimated the median cost per woman to be approximately 15,000 euros (19,690 USD). In another five-year prospective cohort study, involving 739 Danish women initiating fertility treatment in 2000 − 2001, in four large public clinics, Christiansen et al. aimed to estimate the costs of ART. This study evaluated frozen/thawed embryo transfer, intracytoplasmic sperm injection, IVF and intrauterine insemination [15]. The study was conducted from the public healthcare sector perspective. Treatment data were abstracted from medical records (and valued in a second step). The total expenditure per treated woman, irrespective of whether the treatment was successful, was estimated to be 6,607 euros. Finally, Peeraer et al. conducted a retrospective cost analysis, from a societal perspective, of 213 women who became pregnant and had a live birth after one or more ART treatment cycles. Based on a university hospital’s information system in Belgium the authors estimated the average cost of a single pregnancy from the start of infertility treatment until birth to be €4,553, €2,883, and €4,713 for ART (IVF/intracytoplasmic sperm injection), consultations, and hospitalisation respectively [16]. A few other studies were economic evaluations, such as cost-effectiveness analyses comparing ART with expectant management for unexplained subfertility [17], freeze-only with fresh embryo transfer [18], and gonadotrophin therapy with clomiphene citrate [19].

The objective of this study was to analyse the size and distribution of infertility treatment expenditures over time, and estimate the economic burden of infertility treatment per 10,000 women aged 18 − 50 in France, based on a medico-administrative database, using a difference-in-difference method.

## Methods

### Database

We investigated a 1/97th random sample of the National Inter-Scheme Information System on Health Insurance (Système national d'information inter-régimes de l'Assurance maladie, SNIIRAM) covering 98% of the French population, called the Echantillon Généraliste de Bénéficiaires (EGB) [20]. The EGB is a medico-administrative database which constitutes a representative sample of the French population in terms of age and gender. It comprises more than 660,000 individuals, whether they receive healthcare or not [20]. The EGB is an open cohort that is continuously updated with new beneficiaries and newborn infants. The EGB contains exhaustive information on all outpatient care performed (volumes) and reimbursed (values) by the national health insurance. It also contains information on patient-specific administrative data, such as date of birth, gender, place of residence, and conditions for reimbursement of care (total or partial coverage of insured persons). The EGB is linked to the private and public hospital discharge database (Programme de Médicalisation des Systèmes d’Information, PMSI). The PMSI is a medico-administrative hospital discharge database set up to evaluate the costs of hospital stays according to different Diagnosis Related Groups (DRGs). The PMSI provides exhaustive information on public and private hospital care in France, such as diagnoses (coded by physicians, using the International Classification of Diseases, 10th version (ICD-10)), underlying comorbidities, dates and lengths of stay. The PMSI also contains information on outpatient visits and technical medical acts performed in hospitals, available since 2013.

### Study population

All women who purchased a pharmaceutical treatment used in infertility (see Additional file 1) in 2014 and did not receive any of these treatments in the previous three years (rolling year between 2011 and 2014) were included in the study, at the date of their first purchase. The date of inclusion in the study was the date of purchase of the first infertility treatment in 2014. The study population was limited to women aged 18 to 50 in 2014, who were living in mainland France and were affiliated to the general scheme, and who did not have a long-term disease (defined as a disease in which the severity and/or the chronicity require a long-term and particularly costly treatment), including cancer, during the 3-year follow-up period after the beginning of inclusion (rolling year between 2014 and 2017). When a woman gave birth, her follow-up was censored at the time of early pregnancy measured by the estimated date of the first day of pregnancy.

### Study design

A self-controlled before-after analytic design was used to evaluate the economic burden of infertility. The difference of expenditure in overall healthcare resource utilisation was calculated for each patient, by semester. The healthcare resources considered were hospitalisation (private and public), pharmacy, consultations, technical acts, biology, others (including nursing care, midwifery, physiotherapy, dental care, transportation, medical devices and services, and cash benefits). They were considered during the 2 semesters preceding the date of inclusion (pre-treatment) and the following 6 semesters (post-treatment) (rolling year between 2013 and 2017). To control for any changes over time in healthcare resource utilisation independent of infertility, we selected a group of matched controls and conducted the same expenditure assessment 2 semesters before and 6 semesters after a matched index date. Infertility-associated expenditure was derived as a difference-in-difference (DiD), in which the difference between expenditures for patients treated for infertility and non-treated controls were regarded to be associated with the infertility event.

### The matching method

We used the exact matching method to select the control group. The exact matching method is a method that associates one or more controls with identical matching characteristics with the cases (the treatment group) [21]. The control group was constituted in several phases. Firstly, we selected all women aged between 18 and 50 in 2014, who did not receive any fertility treatment between 2011 and 2017, and who were living in mainland France and affiliated to the general scheme. Just like the women treated for infertility, we limited the control population to women who did not have a long-term disease or were not treated for cancer during the follow-up period. Secondly, for each case in the treatment group, controls were selected at random on each of these four variables collected in 2013: age (in six categories), Universal Health Coverage (Couverture Maladie Universelle, CMU) as a dummy variable, the quintile of social deprivation index (Indice de désavantage social, FDep13), the quintile of the Local Potential Accessibility to gynaecologists (Accessibilité potentielle localisée, APL). An individual could benefit from CMU in 2013 if he/she was legally resident in France for more than 3 months and if he/she had resources below a ceiling based on the composition of the household. CMU exempts patients from advance payment of expenses. It is used here as a proxy for individual socio-economic status. FDep13 is an ecological measure that characterises the socio-economic commune in which individuals live [22]. APL measures the spatial adequacy between the supply and the demand for healthcare at the city level [23]. This indicator takes into account access to practitioners based on distance, practitioners’ volume of activity, and service use rates differentiated by population age structure. APL is expressed in terms of full-time equivalent per 100,000 inhabitants.

Controls matched to a case—a woman who gave birth during follow-up—were censored at the time of the case’s early pregnancy. Controls matched who themselves gave birth during the follow-up were not censored.

### Economic analysis

We carried out a DiD regression estimation [24–26] under the assumption that the differences between the groups (cases and controls) would have remained constant without treatment. DiD was implemented as an interaction term between time and treatment group dummy variables in a linear regression model, as follows (see Additional file 2):

1 $$y_{ij}=\alpha+\beta{\text{I}}_i+{\textstyle\sum_{j\neq-1}}\lambda_j{\text{S}}_{ji}+{\textstyle\sum_{\begin{array}{c}j\neq-1\end{array}}}\delta_j{\text{S}}_{ji}\times{\text{I}}_i+\varepsilon_{ij}$$

where *y*_*ij*_ is the total expenditure of *i*^*th*^ woman at the *j*^*th*^ semester;

I_i_ is an indicator of treatment group, case group (I_i_ = 1), or control group (I_i_ = 0);

*S*_*ji*_ represents the *j*^*th*^ semester of the *i*^*th*^ woman for *j* = 1, . . . , 8:

where $t=1,\dots ,48$ is the number of months since January 2013;

We chose the -1 semester (${\text{S}}_{-1i}$, 1 to 6) as the baseline.

$\alpha$ is the average expenditure in the control group at semester -1;

β is the average expenditure (all expenditures combined) differential between cases and controls at semester -1;

${\lambda }_{j}$ is the average expenditure (all expenditures combined) differential between semester j and semester -1, in the control group;

${\delta }_{j}$ is the DiD between cases and controls between semester $j$ and semester -1, i.e. the infertility-associated expenditures.

This model made it possible to calculate the infertility-associated costs in the semester preceding the beginning of treatment (Semester 0) and those in the following 6 semesters, and also the infertility-associated expenditures per woman and per 10,000 women over the 3.5-year follow-up period. Using the DiD method, we also examined the different expenditure items separately: hospitalisation (private and public), pharmacy, consultations, technical acts, biology, others (including nursing care, midwifery, physiotherapy, dental care, transportation, medical devices and services, and cash benefits), and their distribution over time.

By including semester 0 as part of the treatment period, we assumed that this is a period during which women were more likely than usual to seek care because of their fertility disorders (discomfort, anxiety, etiological assessment, etc.).

We used the MIXED procedure in SAS 9.4. In the analysis of response profiles, no specific time trend is assumed. Instead, the times of measurement are regarded as levels of the discrete factor. In order to take into account repeated data related to the same women, we computed the empirical (‘sandwich’) estimator of the covariance to correct for any misspecification of the covariance [27, 28]. The data were assumed to be Gaussian, and their likelihood was maximised to estimate the model parameters.

A societal perspective was adopted. The expenditures (95% confidence interval) were converted into 2020 euros, no discount rate was applied.

### Ethics

Access to the EGB (pseudonymous data) is subject to prior training and authorisation. The EGB was approved by the French National Commission for Data Protection and Liberties (Commission nationale de l'informatique et des libertés, CNIL).

## Results

Among the 615,805 people in the database in 2014, a total of 10,459 participants were included in the study: 556 incident women treated for infertility and following the inclusion criteria were matched with 9903 controls (1:18). More than 60% of the cases were between 25 and 35 years of age (Table 1).

**Table 1:** Characteristics of women

			Cases (*N* = 556)		Matched Controls (*N* = 9,903)		
			%(n)		%(n)		
**Matching variables**							
**AGE**	18–24		10.61	(59)	10.72	(1,062)	NS^a^
	25–30		36.69	(204)	36.57	(3,622)	
	31–35		25.72	(143)	25.47	(2,522)	
	36–40		17.99	(100)	18.18	(1,800)	
	41–42		5.58	(31)	5.60	(555)	
	43–50		3.42	(19)	3.45	(342)	
**APL (quintile)**	1st	(less disadvantaged)	19.96	(111)	20.03	(1,984)	NS
	2nd		19.96	(111)	19.80	(1,961)	
	3rd		20.32	(113)	20.45	(2,025)	
	4th		19.78	(110)	19.54	(1,935)	
	5th	(most disadvantaged)	19.96	(111)	20.18	(1,998)	
**FDEP (quintile)**	1st	(less disadvantaged)	19.96	(111)	20.20	(2,000)	NS
	2nd		19.96	(111)	19.83	(1,964)	
	3rd		20.14	(112)	20.23	(2,003)	
	4th		19.96	(111)	19.72	(1,953)	
	5th	(most disadvantaged)	19.96	(111)	20.02	(1,983)	
**CMU**	No		88.31	(491)	88.93	(8,807)	NS
	Yes		11.69	(65)	11.07	(1,096)	

^a^ Chi-2 test, *NS* Not significant

APL: quintile of the Local Potential Accessibility (Accessibilité potentielle localisée) to gynaecologist (measures the spatial adequacy between supply and demand for care at the city level)

FDEP: quintile of social deprivation index (Indice de désavantage social) (ecological measure that characterizes the socio-economic environment in which individuals in a given geographic area live at a given time based on the percentage of workers in the labour force, the percentage of high school graduates aged 15 and over, the percentage of unemployed in the labour force and median household income)

CMU: Universal Health Coverage (Couverture Maladie Universelle) (allows an exemption from advance payment of expenses. It is used here as a proxy for individual socioeconomic status)

**Fig. 1:**
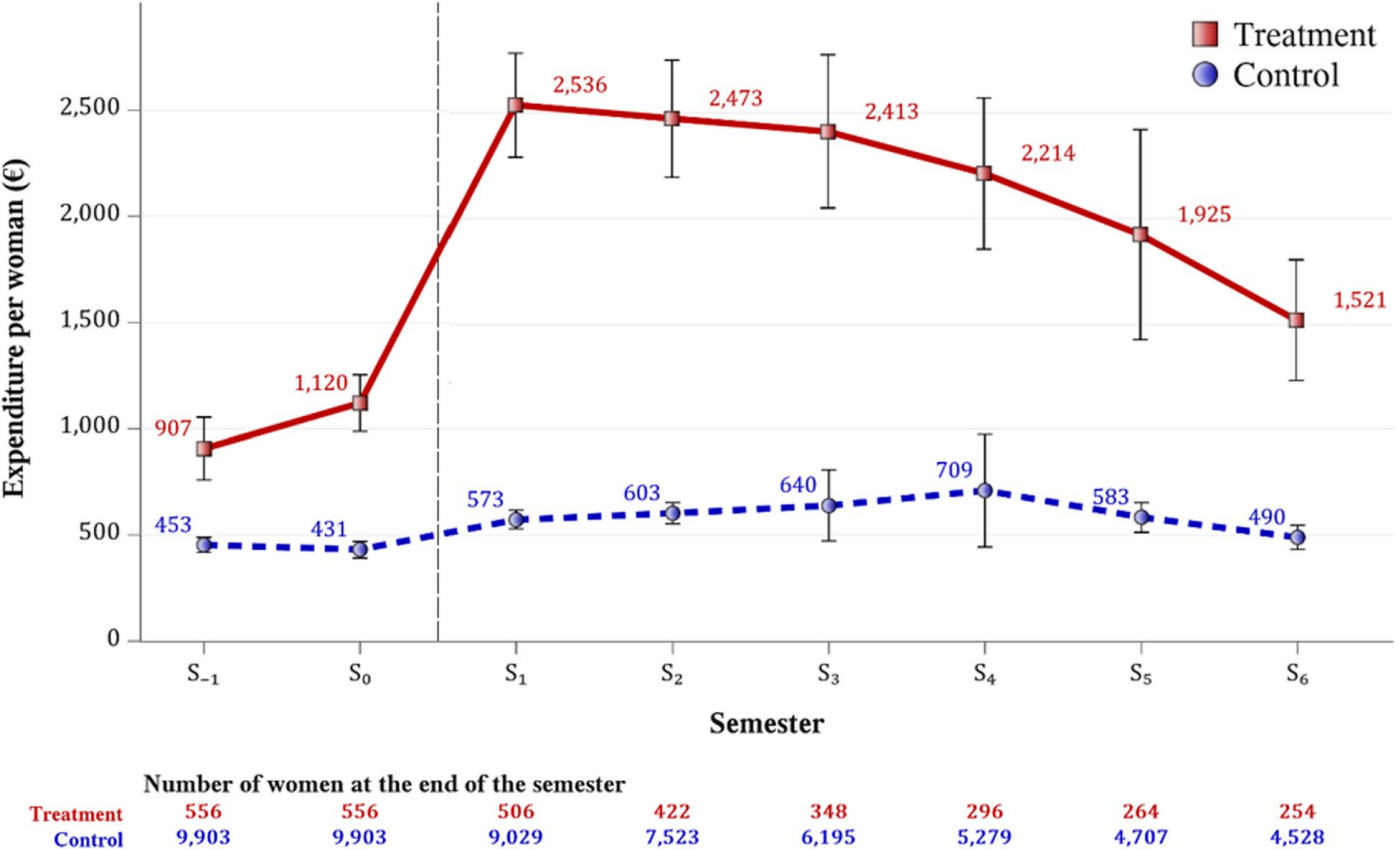
Overall healthcare expenditures (euros) per woman per semester in cases (in red colour) and controls (in blue colour) before and after the first purchase of infertility-related treatment (dashed line)

Three hundred and two (54%) women treated for infertility gave birth during the follow-up period (*see* the number of women remaining in the study at the end of each semester in Fig. 1).

### Descriptive analysis of overall healthcare expenditures

Compared to controls, it can be observed that the overall healthcare expenditures of women treated for infertility increased sharply in the first six months of infertility management (Fig. 1), from 1,120 (IC_95%_: 986 − 1,254) euros in semester 0, i.e. before treatment, to 2,536 (IC_95%_: 2,291 − 2,782) euros in semester 1, reaching a plateau in the two following six months (semesters 2 and 3) and decreasing thereafter until semester 6 to 1,521 (IC_95%_: 1,237 − 1,806) euros. It can also be noted that the healthcare expenditures of women treated for infertility (907 (IC_95%_: 760 – 1,054) euros were already above those of the controls (431 (IC_95%_: 392 − 471) euros), and were on an upward slope before the first purchase of fertility treatment (semester 0).

### Estimation of the infertility-related expenditures using the DiD method

**Fig. 2:**
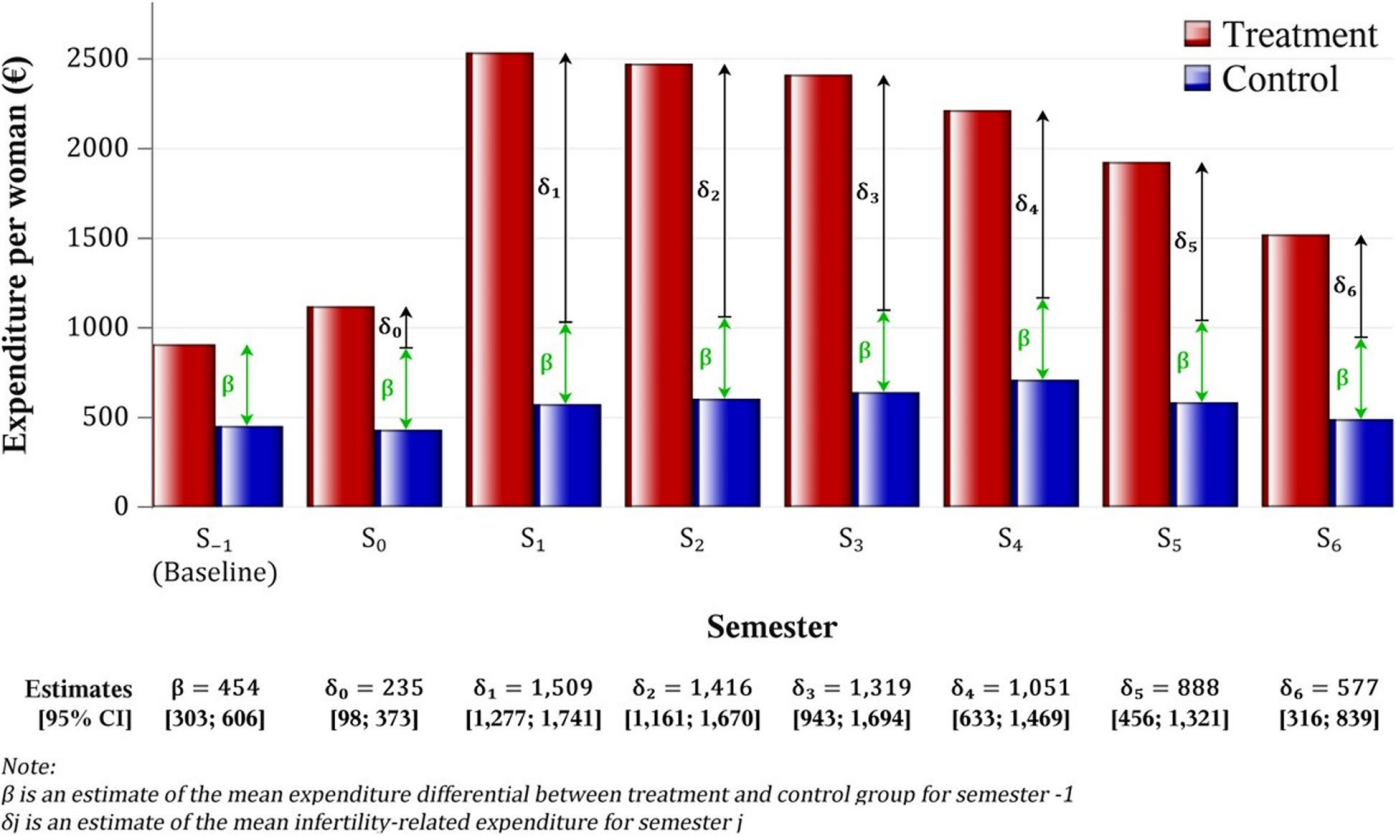
Estimation of the infertility-related expenditures using the difference-in-difference method

The health expenditure specifically related to infertility treatments, and the breakdown of the various expenditure items varied from one semester to another. The infertility-related expenditures (measured by the ${\delta }_{j}$ presented in Fig. 2) increased during the first semester, from 235 (IC_95%_: 98 − 373) euros in semester 0, i.e. before treatment, to 1,509 (IC_95%_: 1,277 − 1,741) euros in semester 1, reaching a plateau in semesters 2 (1,416 (IC_95%_: 1,161 − 1,670) euros) and 3 (1,319 (IC_95%_: 943 − 1,694) euros), and then decreased until semester 6 (577 (IC_95%_: 316 − 839) euros) (Fig. 2).

**Fig. 3:**
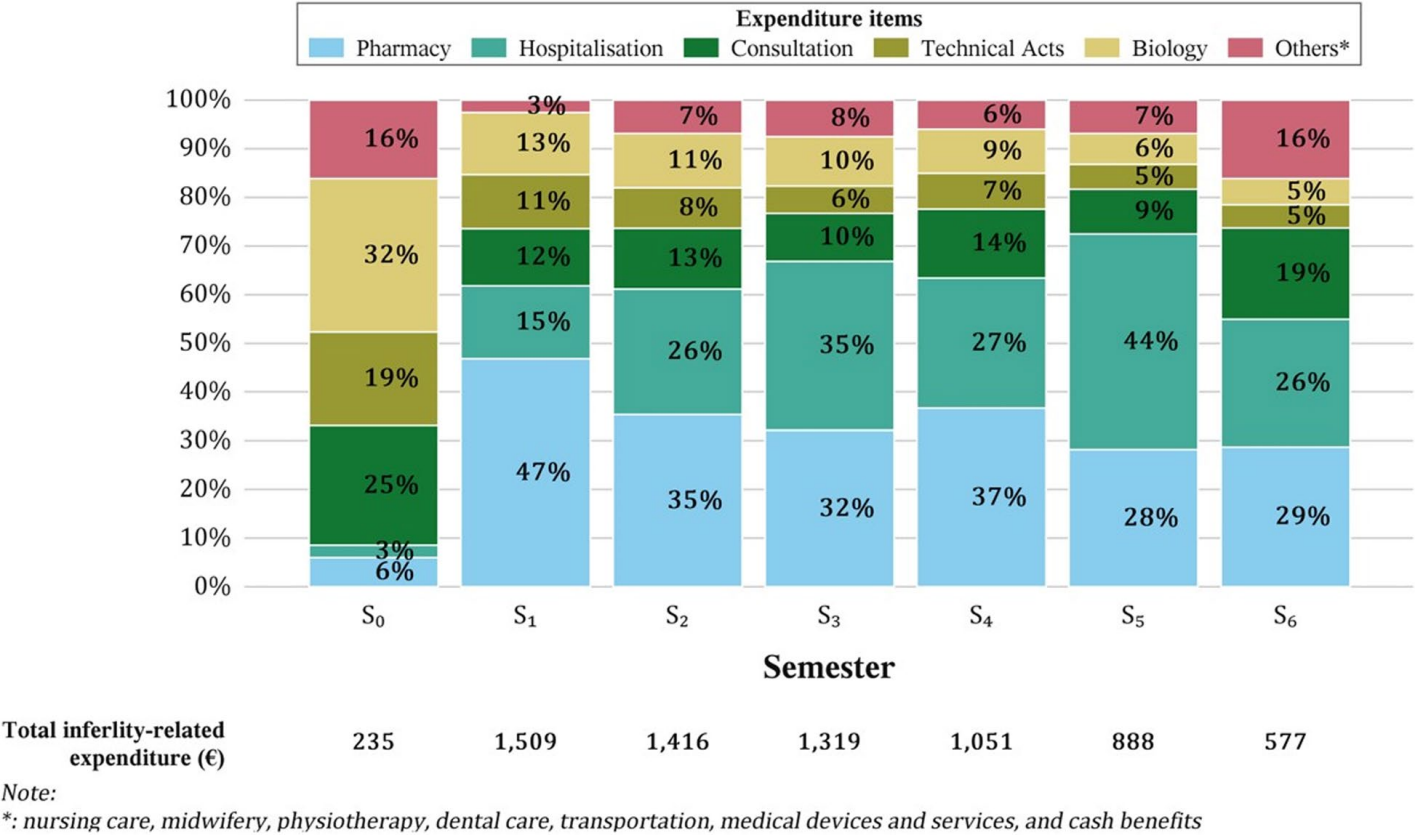
Breakdown of the different expenditure items related to the management of infertility according to semesters, using the difference-in-difference method

While the expenditure related mainly to technical acts, consultations, and biology in semester 0, i.e. before treatment, semester 1 was dominated by expenditure on medicines bought in pharmacy (see Additional file 3) for the 20 most used technical acts between 2014 and 2018). In the subsequent semesters, the share of expenditure on medicines decreased from 47% in semester 1 to 29% in semester 6, while the share of expenditure on hospitalisation rose from 15% in semester 1 to 44% in semester 5 (Fig. 3).

### Estimation of the economic burden of infertility treatment per 10,000 women aged 18 − 50

The average infertility-related expenditure per woman over 3.5 years was estimated to be 6,996 (IC_95%_: 5,755–8,237) euros. We extrapolated the infertility-related expenditures according to the linear model Eq. (1): 70.0 million (IC_95%_: 57.6 − 82.4) euros for 10,000 women observed over 3.5 years (see Additional file 4).

## Discussion

To the best of our knowledge, this is the first study to analyse the size and distribution of infertility treatment expenditures over time, and estimate the economic burden of a cohort of 10,000 women aged 18 to 50 observed over 3.5 years (or less in the event of pregnancy leading to childbirth for women treated for infertility). The average expenditure per woman was estimated to be 6,996 (IC_95%_: 5,755–8,237) euros; the economic burden for 10,000 women was estimated to be 70.0 million (IC_95%_: 57.6 − 82.4) million euros. Healthcare consumption in infertile women increased 6 months before the start of treatment and reached its maximum 6 months after the start of treatment, with a plateau phase during semesters 2 and 3, and then decreased. In the first 6 months after the start of treatment, expenditures were mainly devoted to pharmacological treatment, leaving an increasing share to hospitalisation expenditures during the subsequent semesters.

In all but a handful of countries, cost is an important factor in access to infertility services, including in some developed countries where private insurance prevails [29]. It does not seem that this is the case in France, where infertility treatment is covered at 100% by health insurance, subject to validation of the treatment protocol and only for women up to the age of 43. This coverage is based on the conventional health insurance rate. For those who prefer to go to private hospitals or to gynecologists who charge extra fees, the complementary insurance policies to which the patients have subscribed may cover the cost of care. The rates of coverage vary greatly from one insurance to another. However, although health has no price, it does have a cost that it is important to objectify in order to inform public decision-makers. This is what we are doing in this work. Indeed, in a context of scarce resources, which is not specific to France, the issue of opportunity cost is important to consider. Opportunity cost refers to the loss of benefit that there would be in committing to the financing of a treatment, in this case that of infertility, compared to committing to an alternative treatment offering a better return, in the same or another sector of care.

The changes in the size and distribution of expenditures over time probably reflect first-line and then second line treatments in infertility. Indeed, in case of suspicion of infertility, doctors carry out an initial check-up leading, most of the time, to an ovulation stimulation treatment such as clomiphene citrate or follitropin alpha. Although the cost of Clomid® is not high—around 3 to 7 euros per cycle—, the volume effect is significant. And when one considers the cost of a gonadotropin cycle, which is about 214 to 3,706 euros for Gonal-F® and 183 to 8,920 euros for Fostimonkit®, it is not surprising that the share of expenditures attributed to drugs was high at this stage of the treatment [30]. However, we were surprised by its magnitude. At this stage, it would be interesting to identify precisely the women's care pathways in order to understand the context in which these drugs were prescribed. Were couples counseled about infertility? Were drug treatments alone effective? Did they precede the use of ART? Our study did not aim to answer these questions. However, this result leads us to propose levers for potential action, already widely promoted in the literature. Indeed, after having carried out an assessment of the couple's reduced fertility, in the absence of abnormalities, it is necessary to reassure the couple and to promote a better lifestyle: stop smoking and taking medication, achieve or maintain a satisfactory weight, adopt a healthy diet, have sexual intercourse at an appropriate frequency. This step is sometimes neglected [8].

In a second step, if hormonal treatment is indicated and fails or if assisted reproductive technology (ART) is indicated from the outset, women can benefit from technical procedures such as egg retrieval, for example, which may require hospitalisation costing approximately 1,188 euros [31]. There are few studies in the international literature that have detailed the expenditure items relating to infertility treatment. Even fewer, if any, have looked at their evolution over time in a cohort of treated women. Indeed, according to a systematic literature review published in 2002, a key limitation of the economic studies mentioned by the authors was the failure to provide detailed and disaggregated information on reported expenditures of infertility treatment.

More recently, several studies described in the background have analysed the economic burden of infertility treatment in greater depth [14–16]. The study published by Katz et al. is probably the closest to ours. Unlike our study, the medical resources used, and valued in a second step, were collected both from medical records and interviews conducted with women, when healthcare consumption took place outside a clinic. This may, as the authors themselves point out, have led to some bias in reporting that would make the difference with our study. This does not, of course, explain the cost differential that seems to exist between our two studies, bearing in mind that Katz et al. expressed the results as median while we expressed them as mean (CI). A plausible explanation for this expenditure differential, apart from the fact that the costs of infertility care in the US are usually reported to be high [32], probably has to do with the issue of accounting for expenditures related to pregnancy and childbirth. In contrast with this study, we did not include the period between the beginning of pregnancy and childbirth, which we assumed were a result of a ‘successful’ treatment rather than part of infertility treatment [17]. In particular, it has been shown that the cost of twin pregnancies—more frequent in the case of ART—can be 3 to 5 times that of a singleton pregnancy [33]. In our study, we took into account pregnancies that did not result in childbirth (i.e., miscarriage and medical termination of pregnancy), considering it to be part of infertility treatment. The two other studies cited above focused on ART [15, 16]. Although the results published by Christiansen et al. looks similar to ours, it relates to different forms of expenditure. Indeed, it does not include the costs of hormonal stimulation (for those women who did not need ART), the costs of the couples’ initial visit to their general practitioner before initiating treatment at a specialised public fertility clinic, or the costs for ART treatment prior to the initial treatment at a public fertility clinic. This makes it difficult to compare our results. There is a similar difficulty with the study by Peeraer et al. [16]. The study was conducted on the basis of hospital data and therefore did not include outpatient expenses; the time horizon of the study was different, as women were observed until they became pregnant, and, lastly, the calculations performed included expenditures related to the management of pregnancy and childbirth, which are known to be high [33].

### Strengths and limitations

The main strength of this study is related to the database, which provides detailed and comprehensive cost data, thus limiting the reporting bias inherent in some studies [14]. On the other hand, the EGB does not contain clinical data, which did not enable us to directly attribute the cost of a medical procedure (consultations, ultrasound imaging, other technical acts, etc.) to infertility treatment. But we employed an innovative method—the DiD method—, used for the first time in the study of infertility costs, which has already shown its effectiveness in determining diabetic health expenditure from the same database, for example [34]. The first difference in the DiD was determined for each individual’s expenditures before and after the start of treatment to eliminate individual heterogeneity over time. The second difference was used to control for unobserved temporal effects. In addition, the exact matching method on supply and demand variables enabled us to control some of the confounding factors related to health expenditure, thus avoiding a possible overestimation of expenditures. Another important strength of the study is that we have captured all the costs related to infertility treatment (e.g. consultation with a general practitioner), without them being linked to a local practice, in a population representative of the population of women treated for infertility in France.

This study also has some limitations. First, it is possible that a few women may have had an infertility check-up without ever starting treatment and therefore went undetected. A second limitation of this work is the time-limitation to 3 years after the beginning of the treatment: infertility treatment expenditures were still estimated at 577 euros during the last observed semester (S6) suggesting that there could still be some infertility expenditures after 3.5 years. A third limitation is related to the time interval before the purchase of the infertility treatment which is limited to 2 semesters. Indeed, it is possible that the beta (β) may be slightly overestimated, which would have the effect of underestimating the DiD. Finally, as it is impossible to link the data from two partners, we could not take into account expenditures of the infertility assessment carried out in men.

## Conclusion

This study informs public policy on the economic burden of infertility, from the society perspective, estimated at 70.0 million (IC95%: 57.6–82.4) euros per 10,000 women aged 18 to 50. It also points out the importance of the share of drugs in the expenditure related to infertility treatment. If nothing is done, the increasing use of infertility treatment will naturally lead to increased expenditure. Prevention campaigns against the preventable causes of infertility should be promoted to limit the use of infertility treatments and to contain the related costs. In a context of limited resources, such as in countries with socialized health systems, policymakers must make choices about resource allocation. Investing 10,000 euros in one health sector may prevent the investment of those 10,000 euros in another sector. This study paves the way for future work on the identification of specific care pathways and their valuation, in particular to assess cost effectiveness. This would help guide recommendations on the most efficient management modalities, thus allowing the rationalization of the use of infertility treatments.

## Supplementary Information

**Additional file 1. **

**Additional file 2. **

**Additional file 3. **

**Additional file 4.**

## Data Availability

EGB data is not available (See Ethical approval information and data sharing) however the aggregated data on which the statistical analysis was performed is available upon request.
