# Supplementary material for: The economic burden of infertility treatment and distribution of expenditures overtime in France: a self-controlled pre-post study
Source: BMC Health Serv Res. 2022 Apr 15;22:512. doi: 10.1186/s12913-022-07725-9 (PMC9013027; doi:10.1186/s12913-022-07725-9)
Supplement: Supplementary file 1 — Additional file 1. [file 12913_2022_7725_MOESM1_ESM.docx]

Additional file 1: List of pharmaceutical treatments used to identify incident women treated for infertility
