# Supplementary material for: The economic burden of infertility treatment and distribution of expenditures overtime in France: a self-controlled pre-post study
Source: BMC Health Serv Res. 2022 Apr 15;22:512. doi: 10.1186/s12913-022-07725-9 (PMC9013027; doi:10.1186/s12913-022-07725-9)
Supplement: Supplementary file 2 — Additional file 2. [file 12913_2022_7725_MOESM2_ESM.docx]

Additional file 2: Details of the model used to calculate the infertility-associated expenditure

Let define the indicator variable

$$\text{I}_{i}=\mathbb{1}_{(\text{the} i^{th} \text{woman} \text{is a case})}=\left\{ \begin{matrix} 1 & \text{if the} i^{th} \text{woman} \text{is a case} \\ 0 & \text{otherwise} \end{matrix} \right.$$

where $i=1,2,\ldots,10459$ (556 cases and 9903 control).

For each woman, 48 months of expenditures were observed.
We defined a set of indicators variables which represent the 8 semesters of the study.

$$\left\{ \begin{matrix} S_{-1i}=\mathbb{1}_{(t\in⟦1\ldots6⟧)} & \Rightarrow\text{1 to 6} \\ \text{S}_{0i}=\mathbb{1}_{(t\in⟦7\ldots12⟧)} & \Rightarrow\text{7 to 12} \\ \text{S}_{1i}=\mathbb{1}_{(t\in⟦13\ldots18⟧)} & \Rightarrow\text{13 to 18} \\ \text{S}_{2i}=\mathbb{1}_{(t\in⟦19\ldots24⟧)} & \Rightarrow\text{19 to 24} \\ \text{S}_{3i}=\mathbb{1}_{(t\in⟦25\ldots30⟧)} & \Rightarrow\text{25 to 30} \\ \text{S}_{4i}=\mathbb{1}_{(t\in⟦31\ldots36⟧)} & \Rightarrow\text{31 to 36} \\ \text{S}_{5i}=\mathbb{1}_{(t\in⟦37\ldots42⟧)} & \Rightarrow37 to 42 \\ \text{S}_{6i}=\mathbb{1}_{(t\in⟦43\ldots48⟧)} & \Rightarrow43 to 48 \end{matrix} \right.$$

where $t=1,\ldots,48$ is the number of month since january 2013
We choose the -1 semester ($\text{S}_{-1i}$, 1 to 6) as the baseline

Then for each woman, we compute the total expenditure for each semester. Therefore, we get 8 measurements by woman with a total sample size of $n=83672$ observations $(8\times10459)$.

We choose the -1 semester ($\text{S}_{-1i}$, 1 to 6) as the baseline and we fit the following linear regression model.

$$y_{ij}=\alpha+\beta\text{I}_{i}+\lambda_{0}\text{S}_{0i}+\lambda_{1}\text{S}_{1i}+\ldots+\lambda_{8}\text{S}_{8i}+\delta_{0}\text{S}_{0i}\times\text{I}_{i}+\delta_{1}\text{S}_{1i}\times\text{I}_{i}+\ldots+\delta_{8}\text{S}_{8i}\times\text{I}_{i}+\varepsilon_{ij}$$

$$y_{ij}=\alpha+\beta\text{I}_{i}+\sum_{\begin{matrix} j\neq-1 \end{matrix}} \lambda_{j}\text{S}_{ji}+\sum_{\begin{matrix} j\neq-1 \end{matrix}} \delta_{j}\text{S}_{ji}\times\text{I}_{i}+\varepsilon_{ij}$$

where,

$\boldsymbol{y}_{\boldsymbol{ij}}$ is the total expenditure of $i^{th}$ woman at the $j^{th}$ semester

$\boldsymbol{\alpha}$ is the average expenditure at semester -1, for the controls (1)

$\boldsymbol{\alpha+\beta}$ is the average expenditure at semester -1, for the cases (2)

$\boldsymbol{\beta}$ is the average expenditure differential between cases and controls at semester -1 (3) = (2) - (1)

$\boldsymbol{\alpha+}\boldsymbol{\lambda}_{\boldsymbol{0}}$ is the average expenditure at semester 0, for the controls (4)

$\boldsymbol{\lambda}_{\boldsymbol{0}}$ is the average expenditure differential between semester 0 and semester -1, for the controls (5) = (4) - (1)

$\boldsymbol{\alpha+\beta+}\boldsymbol{\lambda}_{\boldsymbol{0}}\boldsymbol{+}\boldsymbol{\delta}_{\boldsymbol{0}}$ is the average expenditure at semester 0, for the cases (6)

$\boldsymbol{\lambda}_{\boldsymbol{0}}\boldsymbol{+}\boldsymbol{\delta}_{\boldsymbol{0}}$ is the average expenditure differential between semester 0 and semester -1, for the cases (7) = (6) - (2)

$\boldsymbol{\beta+}\boldsymbol{\delta}_{\boldsymbol{0}}$ is the average expenditure differential between cases and controls at semester 0 (8) = (6) - (4)

$\boldsymbol{\delta}_{\boldsymbol{0}}$ is the DID between cases and controls, between semester 0 and semester -1
(9) = (7) - (5) = ((6) - (2)) - ((4) - (1))
(9) = (8) - (3) = ((6) - (4)) - ((2) - (1))

$\varepsilon_{ij}$ are the unobserved random errors, with means zero, representing deviations of the responses from their corresponding predicted means.

We can show that

$\boldsymbol{\alpha+}\boldsymbol{\lambda}_{\boldsymbol{j}}$ is the average expenditure at semester$j$, for the controls

$\boldsymbol{\lambda}_{\boldsymbol{j}}$: is the average expenditure differential between semester$j$ and semester -1, for the controls

$\boldsymbol{\alpha+\beta+}\boldsymbol{\lambda}_{\boldsymbol{j}}\boldsymbol{+}\boldsymbol{\delta}_{\boldsymbol{j}}$ is the average expenditure at semester$j$, for the cases

$\boldsymbol{\lambda}_{\boldsymbol{j}}\boldsymbol{+}\boldsymbol{\delta}_{\boldsymbol{j}}$ is the average expenditure differential between semester$j$ and semester -1, for the cases

$\boldsymbol{\beta+}\boldsymbol{\delta}_{\boldsymbol{j}}$ is the average expenditure differential between cases and controls at semester $j$

$\boldsymbol{\delta}_{\boldsymbol{j}}$ is the DID between cases and controls, between semester $j$ and semester -1
