# Supplementary material for: The economic burden of infertility treatment and distribution of expenditures overtime in France: a self-controlled pre-post study
Source: BMC Health Serv Res. 2022 Apr 15;22:512. doi: 10.1186/s12913-022-07725-9 (PMC9013027; doi:10.1186/s12913-022-07725-9)
Supplement: Supplementary file 3 — Additional file 3. [file 12913_2022_7725_MOESM3_ESM.docx]

Additional file 3: the 20 most used technical acts between 2014 and 2018

| **Number** | **Percent^a^** | **Technical act^b^** |
| --- | --- | --- |
| 1506 | 10.29% | Ultrasound of the female pelvis for ovulation monitoring |
| 1669 | 11.40% | Ultrasound examination of the female pelvis, rectally and/or vaginally |
| 522 | 3.57% | Induction of ovulation by gonadotropins followed by artificial insemination or in vitro fertilization |
| 395 | 2.70% | Non-morphological ultrasound of pregnancy before 11 weeks of amenorrhea |
| 366 | 2.50% | Ultrasound-doppler of the female pelvis for ovulation monitoring |
| 333 | 2.28% | Liquid-based cytopathologic screening examination for cervical sampling |
| 333 | 2.28% | Biometric and morphological ultrasound of a 2nd trimester single pregnancy |
| 331 | 2.26% | Biometric and morphological ultrasound of a uniembryonic pregnancy in the 1st trimester |
| 330 | 2.25% | Supplement for digital archiving of a mammogram or a scan or remnographic examination |
| 309 | 2.11% | Cervicovaginal sampling |
| 301 | 2.06% | Oocyte retrieval from one or two ovaries, transvaginally with ultrasound guidance. |
| 293 | 2.00% | Biometric and morphological ultrasound of a 3rd trimester single pregnancy |
| 232 | 1.59% | Hysterosalpingography |
| 228 | 1.56% | Measurement of the length of the cervical canal of the cervix, by vaginal ultrasound scan |
| 213 | 1.46% | Supplement for intrauterine injection of radiological contrast medium or drug substance |
| 212 | 1.45% | Intrauterine embryo transfer, by vaginal route |
| 168 | 1.15% | Unilateral or bilateral ultrasound of the breast |
| 165 | 1.13% | Intrauterine artificial insemination |
| 143 | 0.98% | Ultrasound and/or Doppler ultrasound to control or monitor the pathology of one or two intra-abdominal and/or intra-pelvic organs or peripheral vessels. |
|  |  |  |
| ^a^ proportion among the total of 14,637 technical acts performed between 2014 end 2018 | | |
| ^b^ according to the French Common Classification of Medical Acts (Classification commune des actes médicaux, CCAM) | | |
|  |  |  |
|  | | |
