# Supplementary material for: The economic burden of infertility treatment and distribution of expenditures overtime in France: a self-controlled pre-post study
Source: BMC Health Serv Res. 2022 Apr 15;22:512. doi: 10.1186/s12913-022-07725-9 (PMC9013027; doi:10.1186/s12913-022-07725-9)
Supplement: Supplementary file 4 — Additional file 4. [file 12913_2022_7725_MOESM4_ESM.docx]

|  |  |  |
| --- | --- | --- |
|  | | |

Additional file 4: Average cumulative expenditures per woman^a^

| **Time since baseline semester*** | **Cumulative expenditures (euros) [IC 95%]** |
| --- | --- |
| 6 months | 235 [98; 373] |
| 1 year | 1,745 [1,440; 2,049] |
| 1.5 years | 3,160 [2,683; 3,637] |
| 2 years | 4,479 [3,810; 5,148] |
| 2.5 years | 5,530 [4,609; 6,451] |
| 3 years | 6,418 [5,301; 7,536] |
| 3.5 years | 6,996 [5,755; 8,237] |
| ^a^ cumulative expenditures calculated using difference-in-difference with baseline semester = semester -1 | |
